# Neuroplasticity Mechanisms in Frontal Brain Gliomas: A Preliminary Study

**DOI:** 10.3389/fneur.2022.867048

**Published:** 2022-06-03

**Authors:** Micaela Mitolo, Matteo Zoli, Claudia Testa, Luca Morandi, Magali Jane Rochat, Fulvio Zaccagna, Matteo Martinoni, Francesca Santoro, Sofia Asioli, Filippo Badaloni, Alfredo Conti, Carmelo Sturiale, Raffaele Lodi, Diego Mazzatenta, Caterina Tonon

**Affiliations:** ^1^Functional and Molecular Neuroimaging Unit, IRCCS Istituto delle Scienze Neurologiche di Bologna, Bologna, Italy; ^2^Department of Experimental, Diagnostic and Specialty Medicine, University of Bologna, Bologna, Italy; ^3^Pituitary Unit, IRCCS Istituto delle Scienze Neurologiche di Bologna, Bologna, Italy; ^4^Department of Biomedical and Neuromotor Sciences, University of Bologna, Bologna, Italy; ^5^Department of Physics and Astronomy, University of Bologna, Bologna, Italy; ^6^Neurosurgery Unit, IRCCS Istituto delle Scienze Neurologiche di Bologna, Bologna, Italy; ^7^Neurology Unit, IRCCS Istituto delle Scienze Neurologiche di Bologna, Bologna, Italy; ^8^Anatomic Pathology Unit, IRCCS Istituto delle Scienze Neurologiche di Bologna, Bologna, Italy

**Keywords:** frontal gliomas, neurosurgery, neuroplasticity, task fMRI, arcuate fasciculus (AF), frontal aslant tract

## Abstract

**Background:**

Pathological brain processes may induce adaptive cortical reorganization, however, the mechanisms underlying neuroplasticity that occurs in the presence of lesions in eloquent areas are not fully explained. The aim of this study was to evaluate functional compensatory cortical activations in patients with frontal brain gliomas during a phonemic fluency task and to explore correlations with cognitive performance, white matter tracts microstructural alterations, and tumor histopathological and molecular characterization.

**Methods:**

Fifteen patients with frontal glioma were preoperatively investigated with an MRI study on a 3T scanner and a subgroup underwent an extensive neuropsychological assessment. The hemispheric laterality index (LI) was calculated through phonemic fluency task functional MRI (fMRI) activations in the frontal, parietal, and temporal lobe parcellations. Diffusion-weighted images were acquired for all patients and for a group of 24 matched healthy volunteers. Arcuate Fasciculus (AF) and Frontal Aslant Tract (FAT) tractography was performed using constrained spherical deconvolution diffusivity modeling and probabilistic fiber tracking. All patients were operated on with a resective aim and underwent adjuvant therapies, depending on the final diagnosis.

**Results:**

All patients during the phonemic fluency task fMRI showed left hemispheric dominance in temporal and parietal regions. Regarding frontal regions (i.e., frontal operculum) we found right hemispheric dominance that increases when considering only those patients with tumors located on the left side. These latter activations positively correlate with verbal and visuo-spatial short-term memory, and executive functions. No correlations were found between the left frontal operculum and cognitive performance. Furthermore, patients with *IDH-1* mutation and without *TERT* mutation, showed higher rightward frontal operculum fMRI activations and better cognitive performance in tests measuring general cognitive abilities, semantic fluency, verbal short-term memory, and executive functions. As for white matter tracts, we found left and right AF and FAT microstructural alterations in patients with, respectively, left-sided and right-side glioma compared to controls.

**Conclusions:**

Compensatory cortical activation of the corresponding region in the non-dominant hemisphere and its association with better cognitive performance and more favorable histopathological and molecular tumor characteristics shed light on the neuroplasticity mechanisms that occur in the presence of a tumor, helping to predict the rate of post-operative deficit, with the final goal of improving patients'quality of life.

## Introduction

Neuroplasticity can be defined as the ability of the nervous system to respond to intrinsic or extrinsic stimuli by reorganizing its structure, function, and connections (1). It is a central theme in neuro-oncology and is currently receiving increased attention (2). Several previous studies have suggested that the brain is capable of remodeling itself in the presence of glioma, and it is believed that these changes occur continuously throughout life (3).

Surgery in the proximity of eloquent brain regions for language is burdened by a significant risk of post-operative speech impairment, up to complete aphasia. Therefore, the most accurate pre-operative localization of these areas and their relationship with the tumor is needed to achieve the maximal safe resection, which consists of the removal of the glioma as largest as possible with minimal risks of permanent language deficits in order to ensure a satisfactory patients' quality of life (4, 5).

Although not all brain regions functional for language have been fully delineated and mapped; in the adult healthy brain, hemispheric lateralization and specific eloquent areas have been well-defined (6). Indeed, since the pioneeristic studies by Broca and Wernicke, and afterward confirmed by multiple other investigations, three main brain areas have been identified: the inferior frontal lobe, the superior temporal lobe, and the insula (7–9).

Pathological brain processes, such as intra-axial tumors, may induce cortical reorganization over time, due to plasticity phenomena (10–14). However, these mechanisms are not fully explained. Shedding light on the potential neuroplasticity of a given brain structure or network may be fundamental for the neurosurgical procedure. During the preoperative period, compensatory brain reorganization may limit/delay the severity/onset of functional deficits, and similarly, postoperative plasticity can help recover from potential deficits associated with the possible removal of still-functional brain areas [e.g., (15, 16)]. In this contest, a complete neuropsychological assessment plays an important role, helping to determine the extent of the “initial” neurological deficit and subsequently document the degree of functional recovery (3).

At the molecular level, single-cell genetic and epigenetic analysis, one study (17) revealed that early genetic alterations were associated with DNA methylation modifications which cause altered cellular states that overcome cell stress, increase cellular plasticity, and ultimately enhance treatment resistance.

This study aimed to evaluate functional compensatory cortical activations in patients with frontal brain gliomas during a phonemic fluency task and to explore correlations with cognitive performance, white matter tracts microstructural alterations, and histopathological and molecular characterization with the main goal of defining a surgical paradigm that may maximize the extension of tumor removal when contralateral compensatory mechanism is present.

## Materials and Methods

### Participants

A total of 15 patients (mean age: 46.24 ± 16.37 years; 10 M; 14 right-handed and one ambidextrous, monolingual native speaker Italian) with frontal intra-axial tumors operated in our Institution from September 2019 to June 2021 have been prospectively collected. In all patients with left-sided glioma (*N* = 10), the tumor presented an intimal spatial relationship with the frontal aslant tract (FAT). For six of these patients, the tumor was also in close relationship with the arcuate fasciculus (AF), and for four of them were adjacent to the frontal operculum. Inclusion criteria were as follows: age ≥18 years; the presence of a frontal intra-axial lesion in a language-related area; and planned surgery with resective aim. Exclusion criteria were as follows: absence of histological demonstration of glioma; previous resective brain surgery or radiation or medical oncological treatment; and biopsy (open or stereotactic frameless) not followed by surgery. Demographic and clinical features are reported in Table 1; a frequency map of tumor distribution is reported in Supplementary Materials.

**Table 1:** Demographic and clinical features of patients with frontal intra-axial tumor.

**Patient**	**Age (years)**	**Sex**	**Education (years)**	**EHI**	**Tumor location**	**Tumor volume (cm^**3**^)**	**Tumor grade**	**Histopathology**	**Genetic analysis**	***MGMT*** **methylation status**
F_L_III	58	M	13	0.68	Left -IFG,MFG, SFG	18.08	3	Anaplastic oligodendroglioma	*IDH1 p.R132H; Del1p19q*	Methylated
F_L_IV	40	F	NA	NA	Left -SMA	10.12	4	Glioblastoma	*IDH1-2:WT; TERT C205T*	Unmethylated
F_L_II	30	F	13	1	Left -IFG,MFG, SFG	22.05	2	Diffuse astrocytoma	*IDH1 p.R132H*	Unmethylated
F_L_II	40	M	18	0.89	Left IFG, MFG, Insula	114.09	2	Astrocytoma	*IDH1 p.R132H*	Methylated
F_R_II	38	F	11	1	Right IFG, Insula	29.34	2	Oligodendroglioma	*IDH1 p.R132H; Del1p19q*	Methylated
F_L_IV	41	M	19	0.78	Left -IFG,MFG, SFG	55.85	4	Glioblastoma	*IDH1 p.R132H*	Methylated
F_L_II	38	F	18	0.89	Left- SFG	37.37	2	Diffuse astrocytoma	*IDH1 p.R132H*	Unmethylated
F_L_III	55	M	13	0.84	Left- SMA	46.2	3	Oligodendroglioma	*IDH1 p.R132H; TERT C228T; Del1p19q*	Methylated
F_R_III	54	M	8	0.89	Right- Motor Area	75.66	3	Anaplastic astrocytoma	*IDH1 p.R132H*	Methylated
F_R_IV	47	F	8	0.89	Right- Motor Area (subcortical)	N/A	4	Glioblastoma	*IDH1-2 WT; TERT C205T*	Unmethylated
F_L_II	25	M	11	1	Left- IFG	9.07	2	Diffuse astrocytoma	*IDH1 p.R132H*	Methylated
F_R_III	37	M	18	0.95	Right-SMA	35.92	3	Diffuse astrocytoma	*IDH1 p.R132H*	Methylated
F_L_III	50	M	21	−0.37	Left IFG, Insula	141.47	3	Oligodendroglioma	*IDH1 p.R132H;TERT C228T; Del 1p19q*	Methylated
F_L_IV	65	M	10	1	Left-SMA	37.03	4	Glioblastoma	*IDH1: WT; TERT C228T*	Methylated
F_R_II	76	M	8	0.95	Right IFG, MFG, Insula	116.55	2	Oligodendroglioma	*IDH1 p.R132H; TERT C228T; Del1p19q*	Methylated

*In the first column, patients are labeled according to the tumor's (lobe)_(hemisphere)_(grade). NA, not available. EHI, Edinburgh Handedness Inventory (scores between −1 and −0.5 were considered indices of left-handedness; right-handedness was defined by scores between 0.5 and 1; and scores between −0.5 and 0.5 indicated ambidextrousness). IFG, inferior frontal gyrus; MFG, middle frontal gyrus; SFG, superior frontal gyrus; SMA, supplementary motor area*.

As controls, a cohort of 24 healthy volunteers (mean age: 46 ± 15.9 years; 12 M; 23 right-handed and one ambidextrous) monolingual native speakers of Italian was also recruited for this study (Table 2). Healthy controls were selected from the database of the Neuroimaging Laboratory, designed to collect normative values of quantitative MR parameters for clinical and research purposes.

**Table 2:** Demographic features of healthy control (HC).

**HC**	**Age (years)**	**Sex**	**Education (years)**	**EHI**
*N* = 24	46 ± 16.37	*N* = 12 males	19.50 ± 4.49	*N* = 23 left-handedness
		*N* = 12 females		*N* = 1 ambidextrousness

*Mean ± standard deviations are shown. EHI, Edinburgh Handedness Inventory (scores between −1 and −0.5 were considered indices of left-handedness; right-handedness was defined by scores between 0.5 and 1, and scores between −0.5 and 0.5 indicated ambidextrousness)*.

The study was approved by the Local Ethical Committee (183/2019/OSS/AUSLBO – 19027 (20/03/19), and written informed consent was obtained from all participants.

### Pre-surgery Protocol

#### Neurological and Neurophysiological Evaluation

Initially, the medical history of all 15 patients was collected with specific attention to neurological symptoms and signs, with a focus on possible language impairments, such as aphasia, anomia, paraphasia, or grammatical or syntactic mistakes. In addition, a complete neurophysiological assessment, including somatosensorial, motor, and brainstem auditory evoked responses was performed 24 h before surgery.

#### Neuropsychological Assessment

Years of education, and handedness dominance using the Edinburgh Handedness Inventory (EHI), were calculated in all patients (18). EHI scores between −1 and 0.5 were considered indices of left-handedness, right-handedness was defined by scores between 0.5 and 1, and scores between −0.5 and 0.5 indicated ambidextrousness.

A subgroup of patients (*N* = 10) also underwent an extensive neuropsychological evaluation with a standardized neuropsychological battery that included the following tests: a general screening test [Mini-Mental State Examination MMSE, (19)] and a non-verbal test of general intelligence [Raven's Colored Progressive Matrices CPM'47, (20)]; language skills were assessed using a naming test [The Boston Naming Test short 30-items version, (21, 22)], a Phonemic Verbal Fluency Test (23) and a Category Fluency Test (24). Short-term verbal memory and episodic memory were assessed using the Digit Span forward test (25) and the immediate and delayed recall of Babcock Story Recall Test (BSRT), (26, 27); instead, visuo-spatial short-term memory span and visuo-spatial long-term memory were investigated using, respectively, the Corsi's Block Test (25) and the delayed recall of the Rey–Osterrieth complex figure [ROCF, (28)]. The copy of ROCF further explored the patients' visuo-constructive and planning abilities. Attention and executive functions were first assessed by a general screening test of frontal functions [i.e., Frontal Assessment Battery, FAB, (29); Italian version (30)], then the Stroop test (31), the Digit Span Backward (32), and The Trail Making Test A and B (TMT) (33) were also administrated (Table 3).

**Table 3:** Neuropsychological corrected scores and correlations with cortical fMRI activations and DTI measures.

		**Patients**							***N***	**Score (mean)**	**Sd**	**Normal values cut offs**	**% pathological scores**	**Correlations with right frontal operculum**		**Correlations with left DTI measures**	
		**F_L_III**	**F_L_II**	**F_L_II**	**F_L_III**	**F_L_II**	**F_L_III**	**F_L_IV**	**7**					***P-value***	***r***	***P-value***	***r***
	**Age (years)**	58	30	40	55	25	50	65		46	15.03						
	**Education (years)**	13	13	18	13	11	21	10		14	3.93						
	**Sex**	M	F	M	M	M	M	M									
	**Clinical tests**																
Cognitive screening	MMSE score	28.14	30	26.99	30	30	30	22.11	7	28.18	2.93	≥23.8 (34)	14%				
Non-verbal Intelligence	CPM-47	28.6	36	36	30.3	27.53	36	NA	6	32.41	4.04	≥18.96 (23)	0%				
Executive functions	FAB	NA	NA	NA	18	16.08	18	14.12	4	16.55	1.86	≥13.5 (30)	0%				
	Digit Span Backward	NA	NA	5	NA	5	3	3	4	4.00	1.15	≥3	0%	0.074^*^	0.902		
	Stroop test (RT-sec)	32.28	5.5	14	10.66	14.59	19.55	121.7	7	31.18	40.79	≤ 36.91 (31)	14%	0.074^*^	−0.910		
Verbal memory	BSRT_total items recalled	12.8	14.1	5.13	12.45	12.21	16	7.9	7	11.51	3.73	≥4.75 (26)	0%				
	Digit span forward	5.75	5.5	6.19	6.75	5.6	4.13	4.05	7	5.42	1.00	≥3.75 (35)	0%				
Visuo-spatial memory	Corsi's block test	3.75	5.75	5.5	5.75	4.5	3.5	3	7	4.54	1.15	≥3.75 (26)	29%	0.017^*^	0.954	0.043^*^ (AF)	0.899
	ROCF_delayed recall	12.85	19	NA	18.25	2.42	20.56	19.1	6	15.36	6.88	≥9.47 (31)	17%				
Language	Associative fluency test	37.1	25.8	26.25	42.8	47.92	25.18	19.68	7	32.10	10.53	≥17.35 (23)	0%				
	Category words fluency test	51.6	47.5	35.88	63	33.5	31.38	17.4	7	40.04	15.08	≥25 (24)	14%				
	BNT-short version	30	30	NA	30		30	28	5	29.60	0.89	≥21	0%				
Attention	Trail making test_A	19.2	40	NA	10	28.92	38.5	146.8	6	47.24	50.10	≤ 93 s (36)	17%				
	Trail making test_B	58.22	82	NA	46	93.2	125.25	484.4	6	148.18	167.04	≤ 282 s (36)	17%				
	Trail making test_B-A	39.02	42	NA	36	63.8	86.75	337.6	6	100.86	117.57	≤ 186 s (36)	17%				
Visuoconstructional abilities	ROCF_copy	36	36	NA	36	36	30.38	35.2	6	34.93	2.25	≥ 28,88 (31)	0%			0.944^*^ (FAT)	0.037

*NA, not available; MMSE, Mini-Mental State Examination; FAB, Frontal Assessment Battery; RT-sec, Reaction time, seconds; BSRT, Babcock Story Recall Test; ROCF, Rey-Osterrieth complex figure; BNT-short version, The Boston Naming Test short 30-items version;*

* *p < 0.05, (FDR) <1*.

Furthermore, the severity of depression and anxiety symptoms were evaluated using Beck Depression Inventory (BDI-II, (37) and the State-Trait Anxiety Inventory [STAI-Y, (38)]. Finally, the Cognitive Reserve Index [CRI, (39)] and The Short Form (39) Health Survey [Sf-36, (40)] were also administrated.

#### Brain MRI Acquisition Protocol

The MRI protocol was performed in all patients within 20 ± 22 days (mean ± standard deviation) from surgery using a high-field Siemens MAGNETOM Skyra 3T MRI scanner equipped with a head–neck high-density (64 channels) array coil.

The MRI protocol included T1-weighted 3D Magnetization-Prepared Rapid Gradient-Echo Imaging sequence [MPRAGE, 176 continuous sagittal slices, 1-mm isotropic voxel, no slice gap, echo time (TE) = 2.98 ms, repetition time (TR) = 2,300 ms, Inversion Time (IT) = 900 ms, flip angle = 9°, acquisition matrix = 256 × 256, pixel bandwidth = 240 Hz, in-plane acceleration factor = 2, duration ~5 min] and T2-weighted 3D fluid-attenuated inversion recovery (FLAIR) sequence (SPACE, 176 sagittal acquisition slices, 1-mm isotropic voxel, no slice gap, TE = 428 ms, TR = 5,000 ms, IT = 1,800 ms, flip angle = 120°, acquisition matrix = 256 × 256, pixel bandwidth = 780 Hz, in-plane acceleration factor = 2, duration ~5 min). In patients, 3D T1-weighted MPRAGE images were also acquired after gadolinium administration (0.1 mmol/kg).

#### Functional MRI (fMRI)

In order to assess hemispheric language laterality, the neural correlates of verbal fluency were elicited via a phonemic fluency task with a block design. In order to ensure good understanding and performance of the required task, all patients underwent, before the MRI exam, a training session to familiarize themselves with the same neuropsychologists (MM and MJR) that administrated the neuropsychological battery.

Functional MRI was based on a 2D T2^*^-weighted gradient echo-planar imaging (EPI) sequence sensitive to blood oxygenation level-dependent contrast. Single-shot EPI sequence (56 continuous axial slices, 2.5-mm isotropic voxel, no slice gap, TE = 37 ms, TR = 735 ms, flip angle = 53°, acquisition matrix = 94 × 94, pixel bandwidth = 2,130 Hz, no in-plane acceleration, multiband acceleration factor = 8, phase encoding AP, duration ~5 min). The block design consisted of alternated resting and active blocks, each lasting 30 s, starting and ending with the resting condition (five resting blocks and four active task blocks in total). The active task blocks were composed of acoustic cues delivered at 5-s intervals through MR-compatible earphones that isolated the background MRI noise. During active cycles of phonemic fluency, the acoustic cue stimulus was a letter of the alphabet, delivered every 5 s. After the presentation of the cue, subjects were prompted to covertly generate (i.e., think about) a noun starting with the given letter. Subjects were previously instructed to generate as many nouns as possible within the time lapse between stimuli but not to generate proper names or names of places (cities/lands/continents). During resting blocks, continuous white noise was delivered, and patients were instructed to lie quietly during the scan without active thinking (41).

### Diffusion Tensor Imaging (DTI)

For tractography analyses a high angular resolution diffusion imaging protocol was acquired with b-value = 2.000 s/mm^2^ along 64 diffusion gradient directions, and five volumes without diffusion weighting, based on a 2D single-shot EPI sequence [87 continuous axial slices, 2-mm isotropic voxel, no slice gap, TE = 98 ms, TR = 4,300 ms, flip angle = 90°, acquisition matrix = 110 × 110, pixel bandwidth = 1,820 Hz, in-plane acceleration factor = 2, multiband acceleration factor = 3, phase encoding anterior–posterior (AP), duration ~8 min]. To correct EPI distortion artifacts in the diffusion-weighted scan, an additional sequence of three null b-value volumes was acquired immediately prior to the full diffusion data set, with the same acquisition geometry and timing parameters but inverted phase encoding [posterior–anterior (PA)].

### Brain MRI Analysis Protocol

#### Functional MRI

The fMRI processing pipeline was created using FSL software (https://fsl.fmrib.ox.ac.uk/fsl/fslwiki) (42). Images were skull-stripped using the FSL-bet function and motion correction was performed with the tool “motion correction of functional images using the linear image registration” (FSL-MCFLIRT). Using the output of FSL-MCFLIRT, an automatic pipeline has been developed for quality control of fMRI images: GE-EPI volumes that are displaced more than 1.5 mm in one of the three spatial directions and rotated more than 1.5° around the three spatial axes with respect to the central volume of the temporal fMRI series, are detected. A successive visual inspection was performed to define the number of volumes and the number of contiguous volumes characterized by this motion. Spatial smoothing was performed using a full width at half maximum (FWHM) Gaussian kernel of 5 mm. High-pass filtering of task-based fMRI time series was performed with a threshold of 60 s. FSL-epi_reg permitted registration between structural and functional images. Language-based fMRI data were processed using the FSL-FEAT GUI (FMRI Expert Analysis Tool) (43). Task and rest cycles in block conditions were convolved with the hemodynamic response function to generate the general linear model (GLM). For each subject, fixed-effect GLM was performed using a threshold of z ≥3.1, and then cluster-extent-based thresholding was used, setting *p* < 0.05.

In order to evaluate a hemispheric laterality index (LI), the fMRI activation regions obtained were masked with bilateral ROIs to evaluate activations in selected language areas. Frontal, parietal, and temporal Regions of Interest (ROIs) were extracted from the cortical Harvard–Oxford atlas: frontal ROIs included the inferior frontal gyrus, pars triangularis, pars opercularis, and the frontal operculum; parietal ROIs included the angular gyrus and the posterior supramarginal gyrus; temporal ROIs included the posterior portion of both superior and medial temporal gyri. fMRI activations were non-linearly registered to the MNI-152 space, using the warp field defined by FSL-FEAT. FMRI activation maps registered to the MNI-152 space were thresholded at Z >3.1 and then masked for each subject using the previously defined bilateral frontal, parietal, and temporal areas. The number of activated fMRI voxels was evaluated within each area. The LI was calculated according to the following formula: LI = (Left – Right)/(Left + Right), where “Left” and “Right” indicate the number of voxels activated within the left and right homologous areas, respectively.

These ROIs were investigated on the basis of previous studies (44, 45), and this analysis aims to robustly extract a laterality activation index in each lobe and quantify the reorganization of brain activity in the presence of tumors.

#### Tractography

Diffusion-weighted images were skull-stripped using the FSL-bet function, image denoising was performed with the MRtrix3-dwidenoise function (https://www.mrtrix.org), using a principal component analysis approach. Susceptibility-related distortions in the EPI acquisition were estimated using the FSL-topup function; subsequently, a combined correction for susceptibility, eddy-current effects, and signal dropout, most commonly induced by subject movement, was performed for the FSL-topup estimates. The FSL-dtifit function was used to model diffusivity along the spatial eigenvectors using the tensor model, obtaining the fractional anisotropy (FA) and mean diffusivity (MD) maps. The tractography pipeline was fully automatized. High-order fiber modeling was used to evaluate crossing fibers, and a probabilistic streamline propagation approach was adopted. ROIs defined in the Montreal Neurological Institute (MNI)-152 space were non-linearly registered (FSL-fnirt function) for subject T1-weighted images. The T1-weighted images were then registered to the diffusion-weighted images using the FSL-epi_reg function, which aligns images, simultaneously correcting for distortions using gray–white intensity contrast. To bilaterally reconstruct the arcuate fasciculus (AF) a previously validated seed target approach was used (46); the same approach was used for the frontal aslant tract, (FAT) considering the Supplementary Motor Area (SMA) as seed mask according to the Harvard–Oxford probabilistic atlas and the Brodmann's area 44 (BA 44) as target mask both thresholded at 25% of subject probability. A midsagittal exclusion ROI was defined at MNI-152 space X = 0.

**Figure 1:**
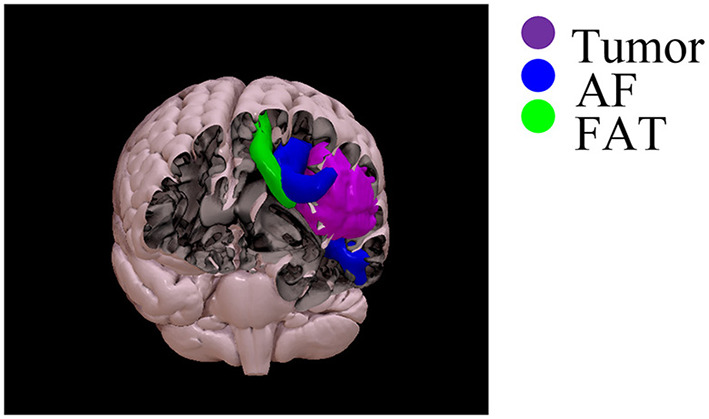
Three-dimensional rendering of the reconstruction of the AF (blue), FAT (green), and tumor segmentation (violet) in the same patient with left frontal glioblastoma grade 4.

Constrained spherical deconvolution diffusion modeling and probabilistic tractography were performed (tckgen ifod2- Mrtrix3) in native diffusion space, into which the tractography ROIs defined in MNI-152 space were non-linearly registered. Tractography results were thresholded at 10% of the maximum connectivity within each voxel, to reduce false-positive artifactual reconstructions. Subsequently, along-tract mapping and statistical calculations were performed in MNI-152 space. AF and FAT tractographic reconstructions and DTI maps were linearly aligned to the MNI-152 space (FSL-flirt, allowing 12 degrees of freedom). A linear registration approach was preferred to preserve the native tract bundle geometry, allowing comparisons of patients and healthy controls in a common space. The SurfIce software (https://www.nitrc.org/plugins/mwiki/index.php/surfice:MainPage) was used for the projection of voxel-wise data onto a surface mesh and to display the reconstruction of tracts and the segmentation of tumor volume in three dimensions (i.e., see an example of one patient with left frontal glioblastoma grade IV) (Figure 1).

#### Tumor Segmentation

A neuroradiologist (FZ, with 10 years of experience in neuroimaging) who was blind to the history of each patient and tumor final diagnosis outlined the ROIs on the unenhanced 3D T2- weighted FLAIR sequence using an open-source dedicated software (ITK-SNAP v3.6; www.itksnap.org). 3D T1-weighted Fast Spoiled Gradient echo acquisition (FSPGR) acquired before and after gadolinium administration, when available, were also displayed alongside FLAIR images to facilitate the segmentation. 3D manual segmentation of the lesions was performed using the three planes at the same time (axial, coronal, and sagittal). Lesions' boundaries were defined according to the extent of the hyperintensity on FLAIR images; inclusion of the enhancing lesion, if present, was confirmed by overlaying the ROIs on the corresponding FSPGR obtained after gadolinium administration.

For subsequent analysis, the segmentation masks were saved in nifti format and the volumes exported in an excel spreadsheet (Table 1).

#### Brain Tumor Surgery

Surgery was performed with a resective aim through a craniotomy targeted at the tumor extension. Intraoperative neurophysiological monitoring was used in all cases, and, when indicated, an awake setting using specific language tests [i.e., Boston Naming Test (21) and selected items of the Aachen Apahsia test (AAT) (47) chosen case-by-case] was adopted (we opted for the sleeping-awake-sleeping technique). Anesthesia was performed consequently, avoiding the use of myorelaxant.

All surgeries were performed with neuronavigational guidance (StealthStation S8 Surgical Navigation System, MEDTRONIC, Louisville, CO, USA) provided by the co-registered data sets of morphological MRI, tractography reconstructions, and phonemic task activations.

Operative and peri-operative complications were considered.

#### Post-operative Course and Follow-Up

Neurological examination, with particular regard to language deficits, was performed at patients' awakening and regularly during hospitalization. An early post-operative brain MRI before and after gadolinium administration (0.1 mmoli/Kg) was performed within 3 days after surgery to assess the extension of tumor removal.

Gross total resection (GTR) was considered if no remnants of the tumor could be detected compared with preoperative MRI scans (i.e., with respect to any residual enhancement), otherwise, resection was considered as subtotal resection, when the residual tumor is <10% of initial mass; and partial when it is larger than 10% of pre-operative volume.

After a case-by-case discussion at the Tumor Board multidisciplinary meeting, adjuvant treatments (radio and chemo-therapies) were started 1 month after surgery, based on the final diagnosis.

Neurological examination was repeated at 15 and 30 days and every 3 months from the discharge; neuroradiological follow-up consisting of a morphological MRI scan was performed every 3–6 months.

#### Tumor Histopathological and Molecular Characterization

Surgical specimens were formalin-fixed and paraffin-embedded (FFPE) according to routine procedures. The diagnosis was assessed by one neuropathologist (S.A) according to 2016 WHO classification of tumors of the central nervous system (48).

Immunohistochemistry was performed in an automated stainer (Ventana, Tucson, AZ, using Ventana purchased pre-diluted antibodies): antibodies anti-GFAP (clone EP672Y, Cell-Marquez), anti-Olig2 (clone EP112, Cell-Marquez), anti- synaptophysin (clone MRQ-40, Cell-Marquez), anti-BRAF V600E (clone VE1, Roche), anti-CD34 (clone QBEnd/10, Roche), anti-IDH1 R132H (Clone H09, Dianova), anti-ATRX (polyclonal, Sigma), anti-p53 (clone DO-7, Roche) were used. Ki67 labeling index (clone 30–9, Ventana Medical Systems Inc, Tucson, AZ, US) was evaluated by counting at least 1,000 neoplastic cells.

DNA from FFPE tissues was purified by the NucleoSpin Tissue kit (Macherey-Nagel, Germany), following the instruction of the provider. Mutational analysis of *IDH1* (exon 4), *IDH2* (exon 4), *H3-3A* (exon 1), and *TERT* (promoter) was performed using locus-specific amplicon libraries with tagged primers based on Nextera™ sequence as previously described (49, 50). Each Next Generation Sequencings (NGS) experiment on MiSEQ (Illumina, San Diego, CA) was designed to obtain a depth of coverage ≥1,000 ×. FASTQ files were processed in a Galaxy Project environment (51), with hg38 as a human reference genome, and mutations were visualized using BWA (Burrows-Wheeler Aligner) and IGV (Integrative Genomics Viewer). *MGMT* DNA Methylation analysis was performed by treating the genomic DNA (50–500 ng) with sodium bisulfite using the EZ DNA Methylation-Lightning Kit (Zymo Research Europe, Freiberg, Germany) according to the manufacturer's protocol. DNA methylation was evaluated using targeted bisulfite NGS for *MGMT* promoter (coordinates on chromosome 10: 129466810- 129467529, reference hg38), generating libraries with the same approach for mutation analysis. FASTQ files were processed in a Galaxy Project environment using BWAmeth and MethylDackel, taking the human GRCh38/hg38 as the reference genome (52).

Identification of the 1p/19q allelic status was obtained using a dual-color FISH analysis and an Olympus BX61 epifluorescence microscope: for each case, at least 100 neoplastic nuclei were counted, and the copy numbers of 1p36/1q25 and 19q13/19p25 were recorded for each nucleus.

### Statistical Analyses

Paired sample *T*-Tests or independent sample *T*-Tests were used to compare, respectively, measurements are taken from the same group of cases (i.e., right *vs* left fMRI activations) or to compare data (i.e., DTI and histopathological and molecular data) between groups. An adaptive significance threshold was applied using the Benjamini–Hochberg false discovery rate (FDR) procedure to account for multiple comparisons (53).

In addition, correlations between fMRI activations, neuropsychological data, DTI measures (i.e., for the AF and FAT), volume, and histopathological and molecular data were calculated using the Pearson correlation coefficient, again accounting for multiple comparisons using the Benjamini–Hochberg false discovery rate, setting the FDR to 0.1 since in our study the number of subjects was <20 (54). All statistical analyses were performed using IBM SPSS vs.27 and Matlab 2021b Bioinformatics Toolbox functions.

## Results

Ten patients presented seizures, followed by language impairment and one with transitory mild aphasia, while the remaining were incidental findings in three asymptomatic patients and in one with gait disturbances. Surgery was performed in an awake setting in eight cases and was uneventful for all but three patients. One developed a surgical field hematoma, causing hemiparesis, which required a re-intervention with the prompt recovery of neurological deficit in the following days. Another presented a post-operative seizure, that was controlled with anti-epileptic pharmacological treatment. The last had a transitory hemineglect of the contralateral side, which spontaneously recovered in a few weeks. GTR was achieved in 11 cases and in 4 a remnant inferior to 20% of the initial volume was detected at post-operative MRI. No patients developed language deficits in the post-operative time, and the one with pre-operative transitory aphasia improved after surgery.

### Cortical Activation Patterns at Functional MRI

**Figure 2:**
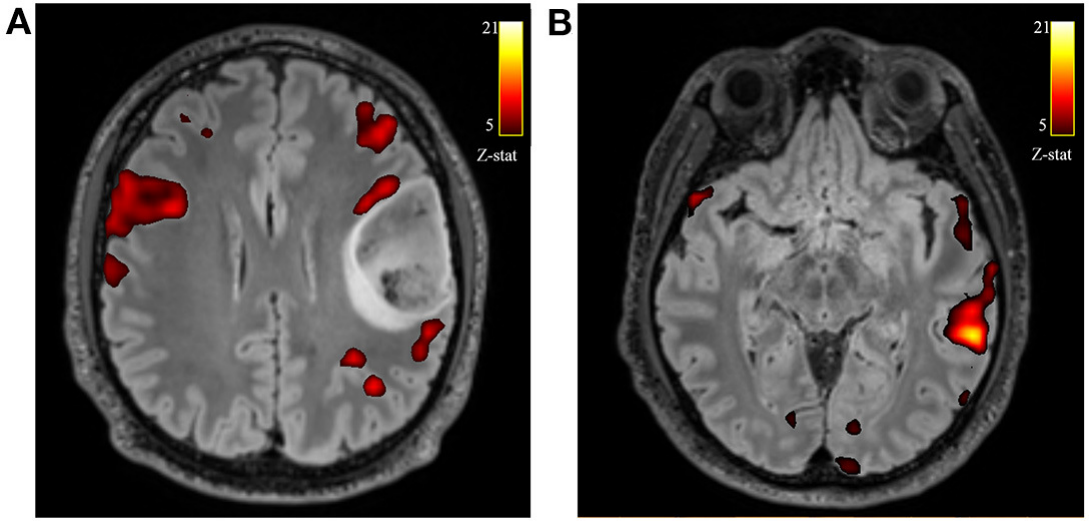
Axial views of the T2-w FLAIR image superimposed the fMRI phonemic fluency fMRI activation. Example of one patient with left frontal glioblastoma grade 4, showing the recruitment of contralateral compensatory activation of right frontal operculum **(A)** and canonical temporal activation on the left hemisphere **(B)**.

All patients during phonemic fluency task fMRI showed left hemispheric dominance in temporal regions (mean voxel on the left = 3835.08 ± 2624.22; mean voxel on the right = 3243.08 ± 2526.33; LI = 0.13) and parietal regions (mean voxel on the left = 3643.69 ± 2956.89; mean voxel on the right = 3164.92 ± 2874.19; LI = 0.09). On the other hand, we found a slightly right hemispheric dominance in frontal regions, specifically in the frontal operculum (mean voxel on the left = 1321.85 ± 961.12; mean voxel on the right = 1552.62 ± 707.99; LI = −0.16), although not significant, when considering the whole group of patients. Instead, when considering only those patients with tumors located on the left side (N = 10) this difference increases, showing significantly higher activation on the right frontal operculum compared to the left (mean voxel on the left = 1097.78 ± 974.29; mean voxel on the right = 1479.11 ± 682.19; LI = −0.27; FDR adjusted *p* < 0.1) (Figure 2). No significant differences were found when considering only those patients with tumors located on the right side (*N* = 5).

Moreover, right frontal operculum activations for left-sided glioma showed significant correlation with neuropsychological performance, specifically with verbal memory (i.e., Digit Span Forward Test, *p* = 0.074; *r* = 0.902), visuo-spatial short-term memory (i.e., Corsi's Block Test, *p* = 0.017; *r* = 0.954) and executive functions (i.e., Stroop Test – time-, *p* = 0.074; *r* = −0.910).

No correlations were found between the left frontal operculum and cognitive performance.

### White Matter Fiber Tracts' Microstructural Features

In order to evaluate to which extents frontal gliomas affect the surrounding neural fibers' integrity, patients' microstructural parameters (MD and FA) of AF and FAT were compared to those of healthy controls. Statistical analyses were conducted by subdividing the patients' samples into two subgroups according to the glioma's localization (left hemisphere, *N* = 10; right hemisphere, *N* = 5).

Independent samples *T*-test (FDR correction, adjusted <0.1) comparing patients with left-sided glioma with controls, showed alterations of left AF microstructural parameters: FA (patients' mean = 0.424 ± 0.019; healthy controls = 0.450 ± 0.024; *p* = 0.021) and MD [patients' mean = (0.607 ± 0.019) 10^−3^ mm^2^ s^−1^; healthy controls = (0.586 ± 0.019) 10^−3^ mm^2^ s^−1^; *p* = 0.021] values. Similarly, MD values of the patients' left FAT showed higher values compared to controls (*p* = 0.021).

**Figure 3:**
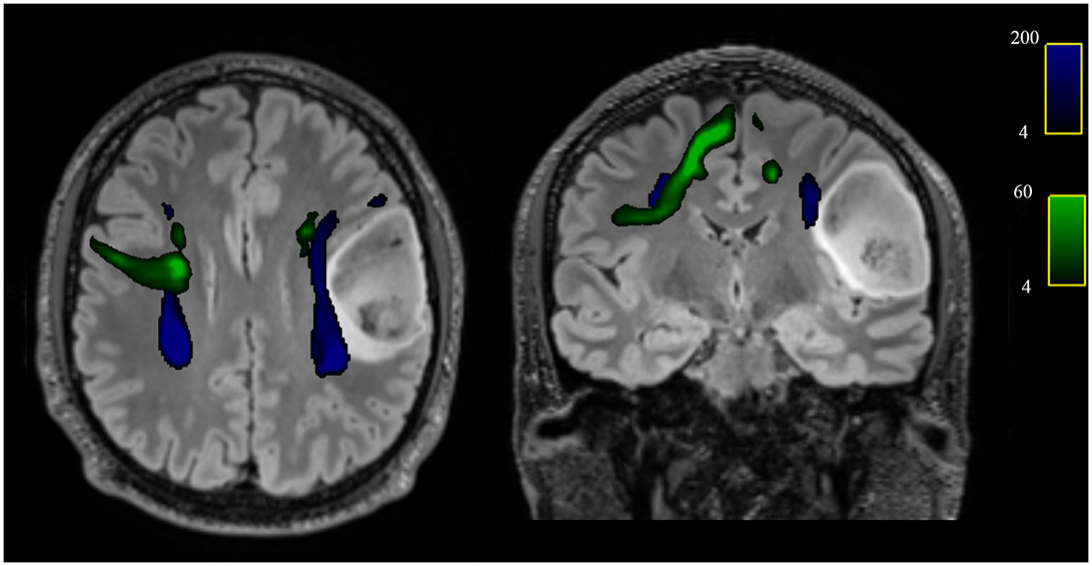
Axial and coronal views of the T2-w FLAIR image with superimposed the reconstruction of the AF (blue) and FAT (green) of one patient with left frontal glioblastoma grade 4, showing the spatial relationship between the tumor and tracts and in particular the displacement of both left AF and FAT.

In addition, comparing the subgroup of patients with right-sided glioma to the controls, a reduction of FA and an increase of MD values were detected (all FDR adjusted *p* < 0.1) in right AF and in right FAT (*p* = 0.027 and *p* = 0.001, respectively) (Table 4 and Figure 3).

**Table 4:** Comparison of DTI parameters between patients and healthy controls.

**DTI features**		**HC (*****N*** **= 24)**		**LG patients (*****N*** **= 10)**		**LG patients vs. HC**	**RG patients (*****N*** **= 5)**		**RG patients vs. HC**
		**Mean**	**Sd**	**Mean**	**Sd**	***P-value***	**Mean**	**Sd**	***P-value***
Left FAT	*MD*	0.594	0.020	0.637	0.072	0.021^*^	0.598	0.038	*NS*
	*FA*	0.404	0.025	0.385	0.053	*NS*	0.404	0.034	*NS*
Left AF	*MD*	0.586	0.019	0.607	0.019	0.021^*^	0.595	0.030	*NS*
	*FA*	0.450	0.024	0.424	0.019	0.021^*^	0.427	0.037	*NS*
Right FAT	*MD*	0.593	0.022	0.602	0.014	*NS*	0.668	0.076	0.001^*^
	*FA*	0.405	0.023	0.389	0.027	*NS*	0.340	0.050	*NS*
Right AF	*MD*	0.586	0.020	0.589	0.016	*NS*	0.671	0.106	0.027^*^
	*FA*	0.433	0.033	0.415	0.030	*NS*	0.385	0.075	*NS*

*FAT, frontal aslant tract; AF, arcuate fasciculus; MD, mean diffusivity values (10^−3^ mm^2^ s^−1^); FA, fractional anisotropy values; HC, healthy controls; LG, left glioma; RG, right glioma; sd, standard deviation;*

* *p < 0.05, (FDR) <1. NS, non-significant*.

*FAT, left and right frontal aslant tract; AF, arcuate fasciculus; LG, microstructural features of patient with left glioma; RG, patients with right glioma; HC, healthy controls*.

Correlation analyses were successively conducted between left/right AF and FAT microstructural parameters with neuropsychological data, and significant correlations were only found in the subgroup of patients with left-sided gliomas. Specifically, FA reduction in the left AF was associated with worse visuospatial short-term memory (Corsi Block Test, *p* = 0.04, 3r = 0.899), and FA reduction in the left FAT was associated with worse performance in visuo-constructional and planning abilities (ROCF copy, *r* = 0.944, *p* = 0.037).

### Histopathological and Molecular Characterization

Histopathological and molecular characterization of tumors was achieved considering tumor grade according to 2016 WHO (48) and several biomarkers (i.e., *IDH-1, TERT, MGMT*, DEL1p/19q, Ki-67). Six tumors confirmed to be grade II according to WHO, five were grade III, and four were grade IV, histological diagnosis was astrocytoma in four cases, oligodendroglioma in two, anaplastic astrocytoma in two, anaplastic oligodendroglioma in three and the remaining were glioblastomas. Molecular characterization is reported in Table 1.

Independent sample *t*-tests were run to explore the influence of these parameters on the frontal operculum compensatory mechanisms, white matter tracts integrity, and neuropsychological performance. Results showed that compensatory activations of the right frontal operculum were significantly higher in patient with *IDH-1* mutation (1,704 ± 577,897) compared to wild-type (720 ± 1018.234) (FDR adjusted *p* = 0.067), instead, no association was found with DTI microstructural parameters. A similar difference in the right frontal operculum activation was found between patients with *TERT* mutation in comparison to wild-type (FDR adjusted *p* = 0.031) and in those with deletion of 1p19q (FDR adjusted *p* = 0.008).

No differences in cortical fMRI activation were observed in patients with *MGMT* methylation, Ki-67 values, or different tumor grades, as well as between all these parameters of histopathological and molecular characterization and DTI microstructural alterations.

Significant differences in neuropsychological performance were observed between patients with *IDH-1* mutated gliomas and wild-type. Specifically, patients with *IDH-1* mutation showed better performance in tests that measure general cognitive abilities (i.e., MMSE, FDR adjusted *p* = 0.007), semantic fluency (Category Fluency Test, FDR adjusted *p* = 0.093), verbal short-term memory (Span forward, FDR adjusted *p* = 0.037), and executive functions (FAB, FDR adjusted *p* = 0.037; TMT-B, FDR adjusted *p* = 0.093).

Instead, no significant correlations were found between tumor volumes and right frontal operculum activations, neuropsychological performance or DTI microstructural parameters.

## Discussion

Phonemic fluency is the conventional test used for the detection of frontal lobe dysfunction (55). Converging findings from healthy controls and lesion studies using functional fMRI demonstrate that fluency tasks activate a left-lateralized network of cortical regions (56). In this preliminary study, we demonstrate that patients with frontal brain gliomas during a phonemic fluency task showed compensatory cortical activations of the corresponding regions in the non-dominant hemisphere. In particular, we found that patients with left-sided frontal gliomas, albeit right-handed, showed significantly higher activation of the right frontal operculum compared to the left. This emphasizes the possible pivotal role that the contralateral frontal operculum could play in the neurocognitive adaptive reorganization in patients with brain gliomas. Indeed, our study shows that the results were associated with improved neuropsychological performance in the domains of verbal and visuo-spatial memory and executive functions, while ipsilateral frontal operculum presented no significant association with cognitive performance. This is in line with the suggestions that the frontal operculum, which contains Broca's area, is essential for language and general cognitive functions, and previous studies that examined patients with lesions of the frontal operculum showed a specific association with clinical aphasias (57, 58).

Surgery of gliomas of eloquent areas is challenging for the double aims that it poses to the surgeon. Although its goal should be complete tumor removal, it should avoid neurological deficits to preserve the patient's quality of life. Particularly, frontal gliomas, involving the dominant operculum areas (usually left-sided), are often considered at high risk for resection. It has been argued that compensatory plasticity mechanisms of the brain may help the surgeon to reduce the risk of post-operative deficit, possibly tailoring the surgical resection or, as proposed by some authors, favoring the implementation of these processes by means of specific stimulations also pre- or post-operatively (59).

Besides functional compensatory mechanisms, white matter tracts (i.e., AF and FAT) play also a crucial role in language and general cognitive processing. In this study we found that the presence of microstructural alterations, i.e., reduction of FA values of the FAT, is associated with worse performance in the Rey–Osterrieth complex figure (ROCF), a test that measure visuo-constructive and planning abilities. This result is in line with a recent systematic review that showed the role of the FAT in the visuo-motor process that supports movement planning (60).

Nowadays, histopathological and molecular characterizations are gaining a crucial role in gliomas. Indeed, many glioma entities are defined on the basis of genetic alterations in various driver genes such as *IDH1-2, ATRX, TP53, CDKN2A/B, TERT*, and *H3 K27* (61). Interestingly, we observed that the contralateral compensatory activation of the operculum region was mostly dependent on some molecular parameters of the tumors, such as *IDH-1* mutation, *TERT* mutation, and deletion of 1p19q, instead of the pathological grade of the tumor. This could suggest that those tumors, with a longer natural history and, at least an initial slower growth, could induce more effectively a contralateral cortical compensation, favoring better neuropsychological performances, as we observed for patients with *IDH-1* mutations. The demonstration of such compensatory cortical activation and the association with better cognitive performance would help to clarify neuroplasticity mechanisms that occur in the presence of a tumor and thus predict the rate of post-operative deficit, with the final goal of improving patients' quality of life. One of the main limitations of this study is related to the number of patients enrolled; therefore, inductive statistical techniques (i.e., multivariate analyses and prediction models) are not suitable. In addition, an *ad hoc* pipeline to perform multiparametric advanced analyses that fuse fMRI and DTI data is not available for a small sample size (62). Further studies with a larger sample and follow-ups are needed to complement these encouraging preliminary results and to confirm our ‘proof of concept' study. If confirmed, the present results enhance the role of presurgical fMRI in guiding a more aggressive surgery of frontal lobe gliomas when a contralateral compensation is evident.

## Data Availability Statement

The data presented in this study are deposited in the online repositories and are accessible with the following link: https://www.ncbi.nlm.nih.gov/sra/PRJNA804145.

## Ethics Statement

The studies involving human participants were reviewed and approved by the Local Ethical Committee AVEC (Area Vasta Emilia Centro) 183/2019/OSS/AUSLBO – 19027 (20/03/19). The patients/ participants provided their written informed consent to participate in this study.

## Author Contributions

MMi, MZ, and CTo contributed to conception and design of the study. MMi, MR, and MZ organized the database. MMi and CTe performed the statistical analysis. MMi wrote the first draft of the manuscript. MMi, MZ, CTe, LM, MR, and FZ wrote sections of the manuscript. All authors contributed to manuscript revision, read, and approved the submitted version.

## Conflict of Interest

The authors declare that the research was conducted in the absence of any commercial or financial relationships that could be construed as a potential conflict of interest.

## Publisher's Note

All claims expressed in this article are solely those of the authors and do not necessarily represent those of their affiliated organizations, or those of the publisher, the editors and the reviewers. Any product that may be evaluated in this article, or claim that may be made by its manufacturer, is not guaranteed or endorsed by the publisher.

## Supplementary Material

Supplementary Figure 1 Frequency map of tumor distribution. **(A)** the percentage of tumor localization is shown at level of the basal ganglia. **(B)** the percentage of tumor localization is shown at level of the motor areas. Axial T1 images are displayed in the radiological convention orientation.
