# Supplementary figures and images for: Neuroplasticity Mechanisms in Frontal Brain Gliomas: A Preliminary Study
Source: Front Neurol. 2022 Jun 3;13:867048. doi: 10.3389/fneur.2022.867048 (PMC9204970; doi:10.3389/fneur.2022.867048)

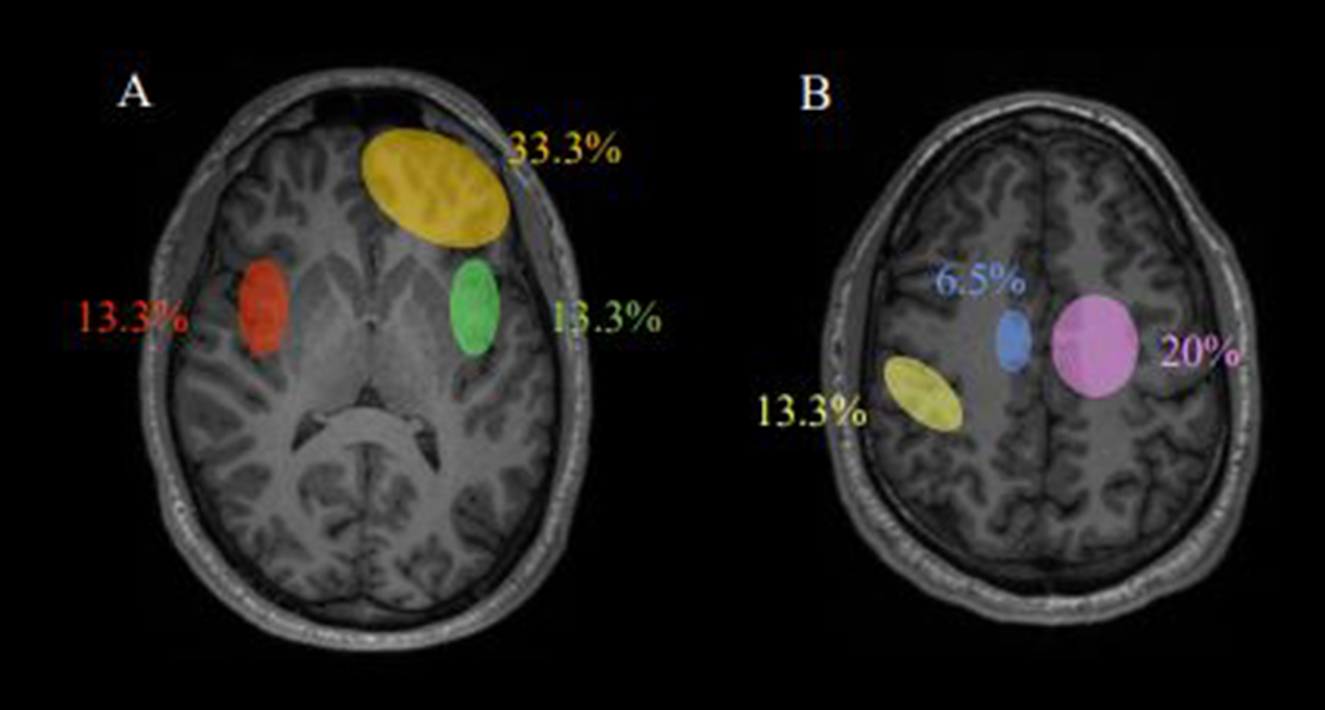

Supplement: Supplementary Figure 1 — Frequency map of tumor distribution. (A) the percentage of tumor localization is shown at level of the basal ganglia. (B) the percentage of tumor localization is shown at level of the motor areas. Axial T1 images are displayed in the radiological convention orientation. [file Image_1.JPEG]
